# : “I’m sorry, Dave, I’m afraid I can’t do that” Part 2

**DOI:** 10.1063/4.0000863

**Published:** 2025-10-27

**Authors:** David R. Rose

**Affiliations:** University of Waterloo, Waterloo, Ontario, Canada

## Abstract

In the WYPT session at the Baltimore ACA meeting in 2023, I instigated a discussion on the use/abuse/future of AI generated text in publications (and elsewhere). One of the main take-aways from that talk was the prospect of AI detecting itself: that is, AI applications that can detect with some level of accuracy and precision whether a piece of text was generated by human or AI. Since then, there have been such routines developed and tested. One (of many) report did a fairly thorough analysis of the most common software: (W.H. Walters, “The Eﬀectiveness of Software Designed to Detect AI-Generated Writing: A Comparison of 16 AI Text Detectors” https://doi.org/10.1515/opis-2022-0158 )

This talk will present some conclusions from that report and pose some questions on how to proceed: Can there be safeguards to distinguish AI text reliably? Can these contribute to defining potential legitimate uses of AI-generated text while still protecting copyright and IP? To what extent might we be able to use AI-generated text to prepare publications, reviews, grant proposals, etc.

